# Participation in Sleep Europe Conferences: Insights From Early Career Professionals

**DOI:** 10.1111/jsr.70378

**Published:** 2026-05-31

**Authors:** Monisha Ravikumar, Michaela Pawley, Anna M. Biller, Lieve van Egmond, Laura Stankeviciute, Matias Rusanen, Daniela Noain, Inês Dias, Iuliana Hartescu, Fran Pilkington‐Cheney, Adriana Michalak

**Affiliations:** ^1^ Early Career Network, European Sleep Research Society Regensburg Germany; ^2^ School of Sport, Exercise and Health Sciences Loughborough University Loughborough UK; ^3^ Institute for Mental Health, School of Psychology, University of Birmingham Birmingham UK; ^4^ Chronobiology & Health, Department of Health and Sport Sciences School of Medicine and Health, Technical University of Munich Munich Germany; ^5^ Department of Psychiatry and Psychotherapy University of Tübingen Tübingen Germany; ^6^ Institute of Neuroscience and Physiology, Sahlgrenska Academy, University of Gothenburg Gothenburg Sweden; ^7^ Department of Technical Physics University of Eastern Finland Kuopio Finland; ^8^ Equality, Diversity and Inclusion Committee, European Sleep Research Society Regensburg Germany; ^9^ Sleep and Neurodegeneration Animal Laboratory, Neurology Department University Hospital Zurich Zurich Switzerland; ^10^ Neurology Department University Hospital Zurich Zurich Switzerland; ^11^ Department of Psychology Nottingham Trent University Nottingham UK; ^12^ ECN Representative, ESRS Board, European Sleep Research Society Regensburg Germany; ^13^ Department of Biomedical, Metabolic and Neural Sciences University of Modena and Reggio Emilia Modena Italy

**Keywords:** conferences, diversity, early‐career, equality, equity, inclusion

## Abstract

Presenting at international conferences is integral for early career professionals' career development by facilitating knowledge exchange, collaboration and visibility. Nevertheless, the barriers and enablers which influence participation among this demographic remain unclear, hampering the establishment of concrete measures to support attendance. This study examined factors affecting participation in European Sleep Research Society (ESRS) conferences, particularly among early career professionals. Ninety‐seven participants (71% female; mean age = 36.5 ± 8.9 years) completed a mixed‐methods online survey, which was distributed among ESRS members and broader professional networks. Early career professionals (81.2%) were defined as MSc/PhD students, early‐career postdoctoral researchers or clinicians. Quantitative data were analysed using descriptive statistics. Reflexive thematic analysis of open‐ended responses enabled identifying key barriers, accessibility concerns and recommendations for inclusivity. Financial barriers were the most cited barrier (66%), followed by caregiving responsibilities (11%), accessibility concerns (10%) and visa difficulties (9%). Although 9% of respondents reported safety concerns related to the host country's environment impaired their attendance, qualitative data indicated that such concerns extended beyond general security issues to anticipated discrimination, exclusion and lack of social or political protection. Eighty‐nine percent indicated that virtual participation would facilitate attendance in situations involving last‐minute illness, visa delays, funding gaps or caregiving responsibilities. These findings suggest that financial and structural inequities are key barriers to participation in ESRS conferences. Whilst hybrid presentation modes, differential registration fees and funding schemes are already in place, greater visibility and promotion of these initiatives is necessary to ensure equitable engagement in sleep conferences.

## 1 Introduction

Participation in scientific conferences is fundamental to academic and professional growth, offering avenues for research dissemination, networking and skills development (Hauss 2020). Conferences also serve as spaces where professionals with diverse backgrounds can engage in cross‐disciplinary dialogue, exchanging expertise and cultivating collaborations that advance both individual careers and collective scientific progress (Vincenta and Donald 2024). For early career professionals (ECPs), including researchers, clinicians, technicians and other healthcare professionals, these events are critical for networking, building visibility in the scientific community and fostering long‐term career progression (Fisher and Trautner 2022; Forsetlund et al. 2021).

Despite the benefits of participation in such events, in‐person attendance remains unevenly distributed across socioeconomic, geographic, career‐stage and disciplinary backgrounds (Velin et al. 2021). Studies examining broader academic and professional conference access have highlighted persistent structural inequities due to financial costs, visa restrictions, caregiving responsibilities and accessibility barriers (Yamashita and Oshimi 2023). These barriers are typically more pronounced for ECPs from low‐ and middle‐income countries (LMICs), whose access to travel funding and institutional support is often limited compared to colleagues from higher‐income nations (Velin et al. 2021).

The benefits of conference attendance are particularly evident for ECPs as they provide essential opportunities for networking, visibility and career progression (Forsetlund et al. 2021; Febria et al. 2022). Previous research has highlighted that greater diversity in conference representation can help address inequities in participation through improving access, engagement and inclusion for underrepresented delegates (Howe et al. 2024). Ensuring diverse representation in conferences, including across career stages and cultural backgrounds, enhances the quality of scholarly debate, as inclusive events support the development and future collaboration of ECPs (Febria et al. 2022). Nevertheless, research often treats ECPs as a homogeneous group, offering narrow insight into how specific barriers disproportionately affect subgroups within early career populations.

The European Sleep Research Society (ESRS) is an international scientific non‐profit organisation dedicated to advancing sleep research and sleep medicine through education, training, scientific meetings and research dissemination (https://esrs.eu/). Through structured initiatives and networking opportunities for ECPs, the ESRS supports professional development of its members, which include researchers, clinicians and students across Europe and beyond (Deboer, et al. 2022). These include early career presentations at the opening ceremony of the biennial Sleep Europe Congress and networking activities such as Early Career Day and the Brain Battle. ECPs constitute a substantial and internationally diverse segment of the ESRS, spanning across disciplines and professional roles within sleep research and clinical practice. The ESRS Early Career Network (ECN) committee was created in 2014 to develop and maintain a peer network of ECPs within the society through organising activities and events to enhance collaboration and participation. Additionally, the Equality, Diversity and Inclusion Committee (EDIC) was launched in 2025 to foster a more equitable and representative professional sleep community, broadening the scope from the Gender Equality Forum (GEF). Although the ESRS has initiated several schemes to support participation in their events, including travel grants for the Sleep Europe Congress and other meetings and courses, fellowships and awards are limited and highly competitive (European Sleep Research Society n.d.). Against the backdrop of rising costs of living and increasingly constrained funding opportunities for ECPs, disparities in conference participation may still persist. Therefore, understanding the barriers and enablers affecting ECPs' engagement is both timely and necessary to inform equity, equality, diversity and inclusion‐focused strategies within the society and beyond.

The present study aims to (1) explore the barriers and facilitators influencing participation in the ESRS Sleep Congress among EC (a group of Early Career Professionals/Researchers & Clinicians) and (2) identify strategies to enhance accessibility, inclusion and equitable engagement for future events. By addressing these aims, this work aligns with ESRS priorities on EC development and reinforces the broader principles of equality, diversity and inclusion in scientific collaboration.

## 2 Methods

### 2.1 Study Design and Procedure

An online survey was administered using Qualtrics between August 22 and November 22, 2025. Participation was voluntary and took between 10 and 15 min. Participants were eligible to take part if they were working in the field of sleep research or clinical sleep medicine. The survey was open to individuals worldwide, including members of the European Sleep Research Society (ESRS), regardless of whether they had previously attended an ESRS Congress. This included individuals who had attended previous congresses, those who had considered submitting an abstract but did not and those who had submitted an abstract but were unable to attend. Recruitment followed a broad, multichannel strategy coordinated through ESRS structures and affiliated organisations to maximise international reach among MSc and PhD students, postdoctoral researchers, educators, clinicians and other professionals involved in sleep research and clinical practice. ECPs were defined as MSc or PhD students or researchers, educators and clinicians within 10 years of obtaining a PhD or MD. The survey was disseminated via ESRS communication channels (including newsletters and social media), ECN and EDIC platforms, ESRS committees and Board members, the Assembly of National Sleep Societies (ANSS) and specialist sleep organisations and using flyers with QR codes at relevant scientific meetings. The questionnaire and recruitment strategies for the study are publicly available via the published workflow, with cross‐links provided for discoverability (Ravikumar et al. 2026a). No formal eligibility screening criteria beyond relevance to sleep research or clinical practice were applied and the survey was administered in English. Ethical approval was obtained from Loughborough University's Research Ethics Subcommittee (project ID: 22836).

### 2.2 Measures

The survey began with introductory questions, measuring sociodemographic characteristics (age, sex, gender, ethnicity, caregiving responsibilities, country of origin) and professional profiles (career stage, employment status, ESRS membership status, language proficiency and country of residence). Migration status (yes/no) was determined based on whether participants recorded a different country of origin from their country of residence. Participants were asked to indicate their current career stage as ‘Master's Student’, ‘PhD Student’, ‘Early‐Career (1‐5 years post‐PhD)’, ‘Mid‐Career (6‐11 years post‐PhD)’ or ‘Senior (11+ years post‐PhD) Post‐Doctoral Research’, ‘Early’, ‘Mid‐,’ or ‘Senior Clinician’, ‘Research Assistant’, ‘Research Nurse’, ‘Sleep Technician’ or ‘Other (please specify)’. For the analysis, we grouped participants according to their career stage: ECPs (master's or PhD students, early‐career clinicians or post‐doctoral researchers), mid‐career professionals (mid‐career clinicians or post‐doctoral researchers) and senior professionals (senior career clinicians or professionals and those who identified as retired). Participants who selected any other response option were categorised as ‘Career stage not specified’. Employment status was assessed with ‘What best describes your current role status?’ with response options: ‘Fixed‐term contract’, ‘Permanent contract’, ‘In clinical training’ and ‘Other (please specify)’. Participants indicated which languages they were proficient in (English, Italian, Spanish, French, German, Other [please specify]) alongside their level. Those who indicated being able to speak English at a C1 level or higher (i.e., C1, C2, Native speaker) and another language at a C1 level or higher were categorised as being able to speak advanced English and another language.

In the main section, participants were asked about their previous conference engagement (number of conferences attended in the previous 24 months, ESRS participation history, the type of presentation[s] submitted and accepted and reason[s] for nonattendance when abstracts were accepted) and barriers and facilitators relating specifically to ESRS conference participation. Participants indicated whether they had previously attended an ESRS Congress (yes/no). This was followed by respondents indicating their most recently attended Congress, which included every event from the 27th Congress in 2024 to the 22nd in 2014, alongside an item for ‘Other event (please specify)’. Participants responded to whether they had submitted an abstract to the ESRS Congress they had attended (yes/no) and whether it was accepted (yes/no/prefer not to say). If participants with rejected abstracts indicated that they did not attend the Congress, they were asked to provide their reasons for non‐attendance and rank them from one (most important) to five (least important). Reasons explicitly included ‘Abstract not accepted’ alongside other potential barriers such as conference fees, visa‐related issues and logistical challenges (e.g., travel restrictions, scheduling conflicts, etc.).

Further structured response options and open‐ended questions were administered to all participants covering financial constraints, visa‐related issues, logistical and scheduling challenges, personal reasons, institutional support, accessibility considerations, language support, safety concerns related to host countries, perceived impact of nonparticipation on career progression and recommendations to improve accessibility, equity and inclusivity (Ravikumar et al. 2026b). Ranking items were completed only by participants who indicated that their abstracts were rejected and who did not attend the conference. Quantitative items captured the prevalence of barriers to conference participation, whilst open‐ended questions explored participants' experiences, accessibility needs and recommendations in greater depth.

### 2.3 Data Analysis

Quantitative data were analysed using SPSS (Version 29, IBM SPSS Statistics, New York, US). In Qualtrics, responses were filtered to include only complete submissions. Of the 254 responses received, 97 were complete and included in the analysis. Descriptive statistics (frequencies, proportions, means and standard deviations) were used to summarise participant characteristics and conference attendance patterns.

Qualitative free‐text responses were analysed inductively using reflexive thematic analysis (Braun and Clarke 2006) in NVivo (Version 14; Lumivero, Burlington, MA, USA). Two researchers (MV, MP) undertook iterative familiarisation, coding and theme development, discussed reflection log interpretations and refined the analytic framework according to the research questions. This process enhanced analytic rigour and consistency, following reflexive thematic analysis recommendations (Braun and Clarke 2006). Word frequency queries and word clouds were used to identify highly salient concepts, which informed subsequent coding but did not determine themes on their own.

The themes were then examined across multiple subgroups, including career stage, caregiving status (i.e., carers for children or individuals with long‐term illness/older adults), gender (i.e., female, male or different to sex assigned at birth), migration status (i.e., working in one's country of origin versus working abroad) and health or accessibility needs (i.e., participants with long‐term health conditions or additional needs). Qualitative free‐text responses were analysed to contextualise and interpret subgroup differences relating to salient barriers and facilitators influencing ESRS Sleep Congress participation. This also encompassed identifying strategies to enhance accessibility, inclusion and equitable engagement for future events. Additionally, based on the emergent themes, the prevalence and distribution of corresponding quantitative variables (i.e., specific barrier‐ and facilitator‐related variables) were examined within the career status subgroups to elucidate patterns between ECPs and other career stages, consistent with the primary focus of the study. Integration of quantitative and qualitative strands was achieved through narrative triangulation (Foss and Ellefsen 2002) and a joint display that juxtaposed numerical findings (e.g., frequency of specific barriers) with illustrative themes and exemplar quotes (Guetterman et al. 2015).

Finally, barriers to attending the ESRS Congress and their associated ranking of importance for non‐attendance were examined among respondents whose abstract was rejected and did not attend the congress. Through quantifying the relative importance of specific barriers and stratifying analyses by career stage, this approach provided complementary insight into the salience and variability of barriers beyond those identified through the qualitative themes.

## 3 Results

### 3.1 Participant Characteristics

Ninety‐seven respondents completed the survey, with a mean age of 36.48 years (SD = 8.85, range 24–70 years; see Table 1). Most identified as female sex (71.1%) and White ethnicity (88.4%), with smaller proportions identifying as Asian (7.4%), Black (3.2%) or mixed ethnicity (1.1%). Around one in nine participants reported caring responsibilities for a child (11.5%) or a person with a long‐term illness or older adult (11.3%). The majority of respondents indicated working in their country of origin (70.8%) as well as being proficient in both English and another language (70.9%).

**TABLE 1:** Participant characteristics.

	*n* = 97
*Sociodemographic variables*	
Age (range from 24 to 70 years); mean (SD)	36.48 (8.85)
Sex; *n* (%)	97
Female	69 (71.1%)
Male	28 (28.9%)
Gender; *n* (%)	95
Female	68 (71.6%)
Male	26 (27.4%)
Different to sex assigned at birth	1 (1.1%)
Ethnicity; *n* (%)	95
Asian	7 (7.4%)
Black	3 (3.2%)
Mixed	1 (1.1%)
White	84 (88.4%)
Carer for child; *n* (%)	96
Yes	11 (11.5%)
No	85 (88.5%)
Carer for someone with long‐term illness or old age; *n* (%)	97
Yes	11 (11.3%)
No	86 (88.7%)
*Professional profile*	
Career stage; *n* (%)	69
Early Career Clinician	14 (20.3%)
Early Career Postdoc	16 (23.2%)
Mid‐Career Clinician	3 (4.3%)
Mid‐Career Postdoc	3 (4.3%)
Senior Career Clinician	2 (2.9%)
Senior Career Professional	4 (5.8%)
Student	26 (37.7%)
Retired	1 (1.4%)
ESRS Membership; *n* (%)	97
Yes	74 (76.3%)
No	23 (23.7%)
*Migration status*	
Country of work same as country of origin; *n* (%)	68 (70.8%)
Country of work different to country of origin; *n* (%)	28 (29.2%)
*Language proficiency*	
English proficiency; *n* (%)	97
Cannot speak	5 (5.2%)
B1 (intermediate)	5 (5.2%)
B2 (upper‐intermediate)	8 (8.2%)
C1 (advanced)	27 (27.8%)
C2 (Proficiency)	34 (35.1%)
Native speaker	18 (18.6%)
Advanced English (≥ C1) and other language; *n* (%)	79
English and another language	56 (70.9%)
Only English	23 (29.1%)

*Note:* ‘Advanced English (≥ C1) and other language’ refers to individuals who recorded being able to speak English at a C1 level or higher (i.e., C1, C2, Native speaker) and another language at a C1 level or higher.

Figure 1 presents participants' overall and ESRS‐specific conference attendance history. Fifteen participants (15.4%) indicated they had not attended any conference in the previous 24‐months, whilst 20 (20.6%) had attended five or more. A significant proportion of respondents attended the most recent ESRS Sleep Congress, Sleep Europe 2024 (*n* = 40; 41.2%); however, many also reported no prior ESRS Congress attendance (*n* = 37; 38.1%).

**FIGURE 1:**
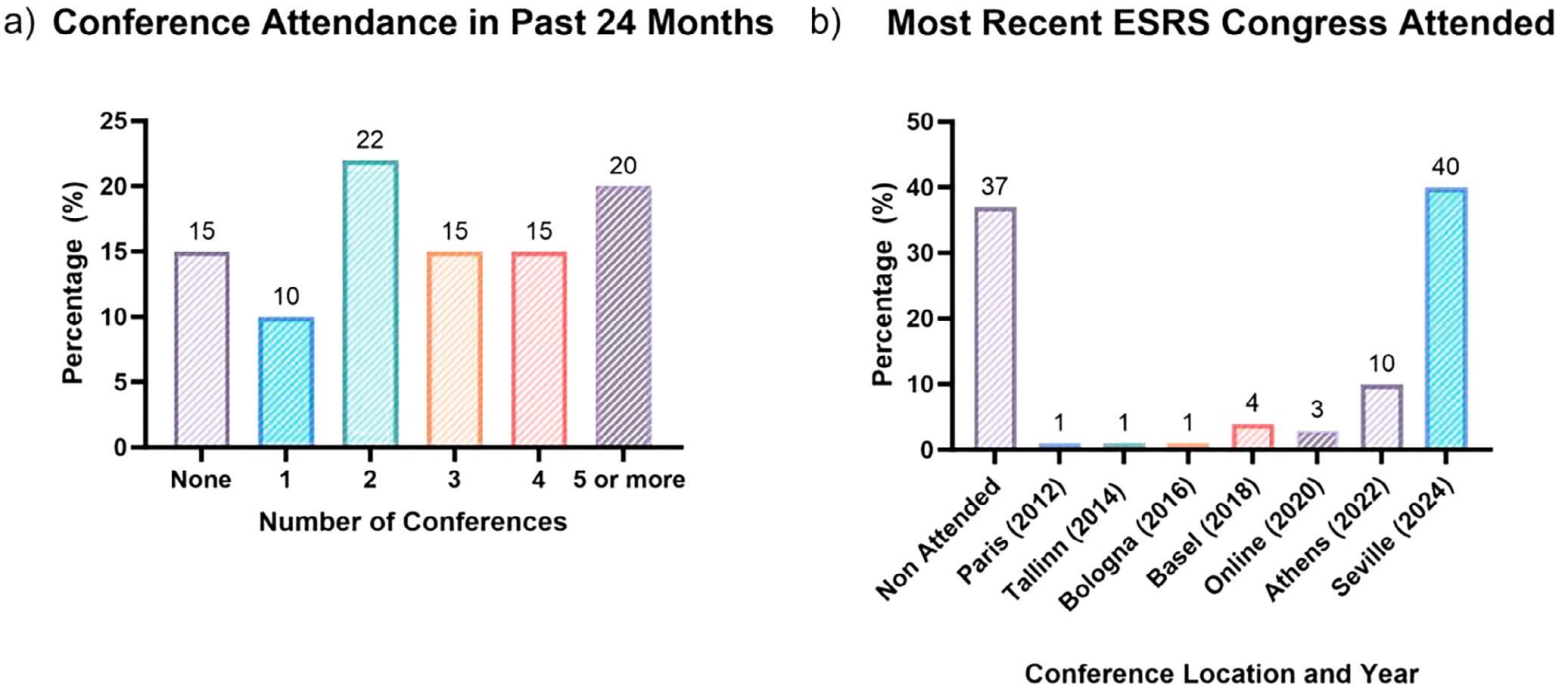
History of overall and ESRS‐specific conference attendance. (a) displays participants' frequency of conference attendance over the prior 24 months and (b) presents the most recent ESRS Sleep Congress attended.

### 3.2 Subgroup Analyses: Integrated Quantitative and Qualitative Findings

Word frequency analysis of free‐text responses identified ‘grants’ (*n* = 102) as the most frequently occurring term, followed by ‘support’ (*n* = 97), ‘travel’ (*n* = 79) and ‘online’ (*n* = 64). Financial‐related words including ‘funding’ (*n* = 52), ‘cost’ (*n* = 49) and ‘childcare’ (*n* = 41), further underscoring the prominence of economic barriers. Logistical and accessibility‐related terms such as ‘time’ (*n* = 29), ‘virtual’ (*n* = 24), ‘accessibility’ (*n* = 11) and ‘visa’ (*n* = 11) were also frequent. When combining singular and plural forms (‘grant’ and ‘grants’), grant‐related references appeared 132 times, reinforcing the centrality of financial assistance in shaping conference participation for participants.

These findings aligned with the five overarching themes which emerged from the reflexive thematic analysis, namely (1) financial and structural barriers; (2) accessibility, disability and safety needs; (3) supervisory and institutional support; (4) flexible and hybrid formats; and (5) career advancement, inclusivity and equity. Barriers to conference participation demonstrated clear patterns by career stage, sex, caregiving status, relationship status, national economic context (e.g., LMICs), migration status (working outside one's country of origin), disability and long‐term health status and additional communication needs (see Table 2) (Figure 2).

**TABLE 2:** Subgroup‐specific barriers to participation.

Theme	Subtheme	Subgroup	Illustrative quotes (participant IDs)	Brief interpretation
Financial and structural barriers	Childcare support for ECPs; grants for childcare; travel support for caregivers	Female carers (Sex = woman; carer for child = yes)	‘*Childcare grants were a BIG HELP… Hybrid participation [also] helped*’. (R005)—‘*Childcare grants, travel and accommodation grants would make my decision to attend much easier*’. (R035)	Females with caregiving responsibilities emphasise that on‐site childcare and dedicated grants are decisive factors for conference attendance.
Accessibility, disability and safety needs	Breastfeeding‐ friendly conference spaces	Female carers	‘*Not a disability, but breastfeeding mothers should be able to breastfeed… I had to run between sessions to breastfeed my baby without proper conditions, standing or sitting on the floor*’. (R005)	Breastfeeding support is described as a major unmet need, despite the presence of a breastfeeding room in Seville, which was not well communicated to participants.
Accessibility, disability and safety needs	Safe locations for single female travellers	Female, single	‘*Locations safe for single women travellers*’ was explicitly requested as a condition for attendance and evening events. (R004)	Safety concerns influence where and how single females feel able to attend and network.
Financial and structural barriers	High registration costs; travel, accommodation and registration expenses; LMICS funding; grants for low‐income researchers	Working in one's country of origin, LMICs	‘*In my country, stipends for early career researchers are very limited… One of the most effective ways to promote attendance… would be to increase financial support and travel grants that fully cover costs*’. (R032)—‘*Because I live in a developing country… it's impossible to attend ESRS events in person*’. (R054)	Participants based in lower‐income settings describe finances as the dominant barrier, often preventing attendance despite strong interest.
Financial and structural barriers	Travel grants; grants	Working abroad (nationality ≠ residence)	‘*Travel grants covering travel and accommodation would definitely help researchers that do not have the budget for travel to attend*’. (R036)—‘*More travel grants for early career researchers, especially where PhD students have no funding*’. (R055)	Researchers working abroad still rely heavily on external travel grants and see them as central to equitable participation.
Accessibility, disability and safety needs	Accessibility challenges on participation; inclusive venue design; platform for wheelchair users; quiet spot	Participants with long‐term health conditions or reduced ability	‘*Conference venues can be chosen that are adapted for people with physical disabilities… interpreters and specialised assistance*’. (R060)—‘*Lighting in auditorium triggered migraines… overcrowding and lack of ventilation… Quiet rooms and more space would help*’. (R055)	Health and sensory issues (mobility, migraines, fatigue) shape how accessible the conference feels beyond baseline step‐free access.
Accessibility, disability and safety needs	Hearing support; sign language services; translation services	Participants with additional needs	‘*Sign language services*’ *and* ‘*written transcripts*’ were highlighted as necessary for inclusivity in talks and poster sessions. (R031, R046)—‘*Translation services, bilingual materials, sign language services*’ were suggested to support diverse attendees. (R023)	Attendees emphasise language and communication access as core to participation.
Financial and structural barriers	Recurring barriers for ECPs; research funding not provided	ECPs (students, early career postdocs/clinicians)	‘*The registration fees are often a lot for ECRs*’. (R001)—‘*My programme only has a very limited budget for conferences… the congress is very expensive*.’ (R020)	ECPs consistently describe registration and travel costs as disproportionate relative to their income or funding.
Supervisory and institutional support	Supervisory and mentorship support	ECPs	‘*More scholarships… There should be some sort of support to attend the conference for those who don't have children*’. (R012) ‘*More information and support around different possible funding methods*’. (R071)	ECPs look to supervisors and institutions for guidance and funding navigation, not just proofreading.
Career advancement, inclusivity and equity	Specific ECP formats and recognition	ECPs	‘*Offer more Rising Stars or similar awards… and research project grants opportunities specifically designed for young researchers*’. (R019) ‘*Please make a conference for early career [researchers]*’. (R011)	ECPs call for dedicated slots, awards and events to facilitate visibility of their work.
Flexible and hybrid formats	Suggestions for alternative presentation formats; virtual oral presentations; virtual participation option for illness	All genders, disabled and non‐disabled	‘*Virtual presentations would be good… Posters prerecorded… presenters join a live Zoom call to answer questions*’. (R018)—‘*Always offer a hybrid participation option with on‐demand replay*’. (R019) ‘*For posters, some congresses* (e.g., *EAN*) *already propose virtual formats of posters with projection on a screen; an option would be to also allow an online presentation (Zoom etc.)*’ (R014)	Across subgroups, participants endorse hybrid and alternative formats as key to inclusion, particularly for those with health, caring or financial constraints.

*Note:* ‘Female’ refers to sex assigned at birth, as reported by participants. ECPs = Early‐career professionals.

**FIGURE 2:**
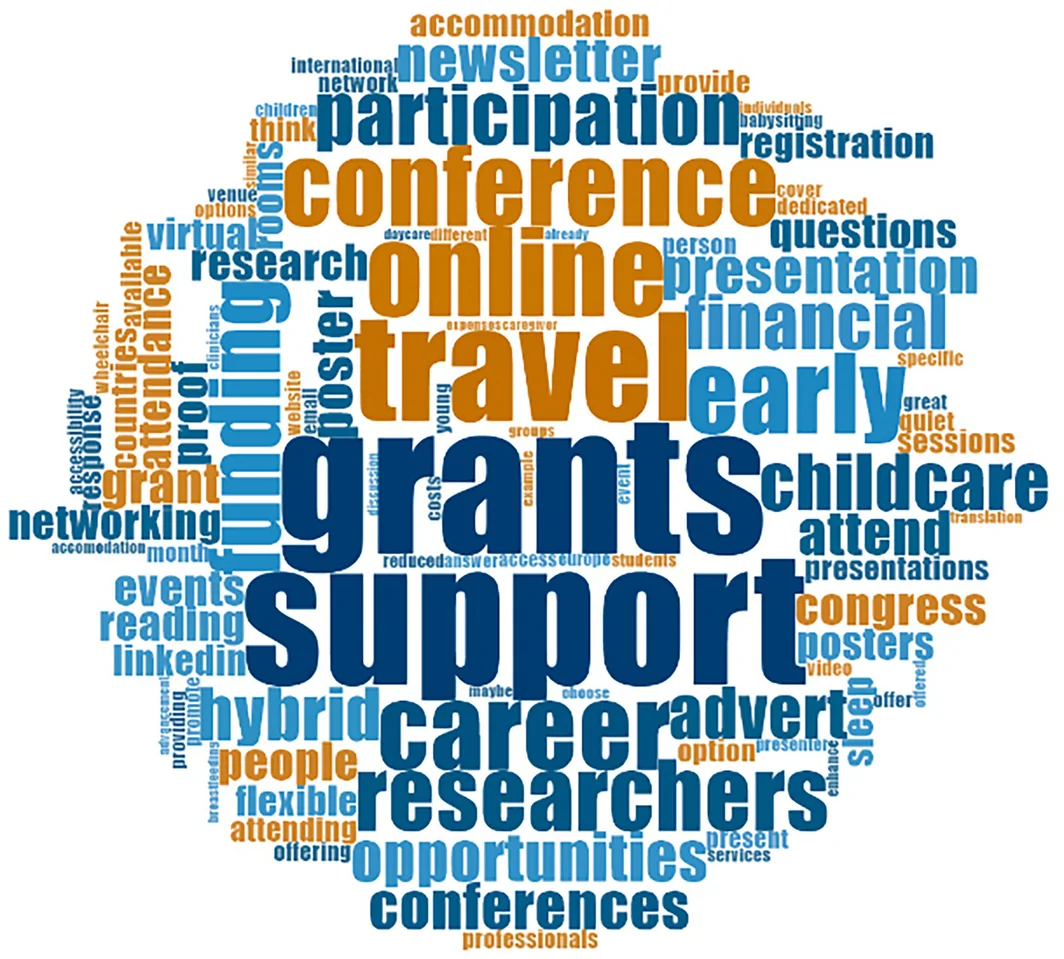
Word frequency count. The frequency of specific terms within participants' free‐text responses across all survey items. The word cloud includes words that were mentioned at least 50 times. Words written in larger font sizes were mentioned more frequently than words in smaller font sizes. The word cloud was generated using NVivo (Version 14; Lumivero, Burlington, MA, USA).

#### 3.2.1 Financial and Structural Barriers

Participants highlighted that attending conferences was ‘*extremely difficult*’ (R036, R061) and ‘*very costly*’ (R047, R077, R086) as PhD stipends often did not extend beyond basic living expenses to cover conference‐related costs, including registration, travel and accommodation. These financial challenges were particularly pronounced for researchers in LMICs, who also reported visa‐related barriers (*n* = 11), including delays, administrative burdens and additional costs. Together, these factors created further challenges in attending international conferences, reinforcing structural inequities in participation. One respondent noted that with a stipend ‘not exceeding 600 USD per month’ it was ‘extremely difficult to afford the costs of international congresses, including registration, travel and accommodation’ (R032). Others quoted ‘cost cost cost cost’ (R087) and ‘*too expensive*’ (R097) as key reasons for non‐attendance and not obtaining ESRS membership. A small proportion of respondents (*n* = 8; 9.2%) reported requiring a visa to attend an ESRS Congress. Among these, two indicated experiencing challenges with the application process (see Table 3). This included an ECP and a career stage unspecified participant.

**TABLE 3:** Visa required to attend ESRS Congress and challenges with the application process.

	Visa required (*n* = 87)		Challenges with application process (*n* = 8)	
	Yes (*n* = 8)	No (*n* = 49)	Yes (*n* = 2)	No (*n* = 6)
ECPs	3	30	1	2
Mid‐career	1	3	0	1
Senior career	1	4	0	1
Career stage not specified	3	12	1	2

*Note:* Challenges with the visa application process only include respondents who were required to apply for a visa.

Abbreviations: Career stage not specified, participants did not indicate the stage of their career; ECPs, student, early‐career clinician, early‐career postdoc; Mid‐career, mid‐career clinician, mid‐career postdoc; Senior, senior career clinician, senior career professional, retired.

In addition to direct financial barriers, some responses attributed limited institutional support and mentorship to further aggravate these challenges. ‘In our country, if you or your institution doesn't have funding for your research then it's not possible to attend the conference because state institutions don't have budgets to cover it financially’ (R075). ECPs noted that further information and support towards alternative funding methods would facilitate attendance. One participant explained: ‘More scholarships, childcare is one option. But there should be some sort of support to attend the conference who doesn't have children. Not every early‐career professionals are parents’ (R012).

#### 3.2.2 Accessibility, Disability and Health

Challenges related to venue layout, lengthy standing periods at poster presentations, sensory environments and physical access emerged as relevant subthemes across all career stages, while specific issues were particularly salient for certain subgroups. Participants with long‐term health conditions or sensory sensitivities reported that ‘lighting in [the] auditorium’ triggered migraines, overcrowding and poor ventilation and called for ramps, elevators, wheelchair accessible stages, quiet rooms and adequate seating as ‘*bare essentials*’ (R055 and R008, respectively). Additional needs such as breastfeeding‐friendly spaces and practical support for attendees who were accompanied by children or dependents under their care were also quoted, particularly among female carers.

Safety and inclusion concerns towards host country political and social contexts were especially salient for single women, LGBTQ+, migrants and ethnic or religious minority respondents. Some disclosed avoiding conferences in countries perceived as hostile to immigrants or LGBTQ+ communities, with one participant stating: ‘I will not attend conferences in countries where the government and people are clearly against immigrants (especially Muslim immigrants), as I would feel a strong sense of isolation from the moment I arrive until the moment I leave the country’ (R083).

In the quantitative analysis, the influence of safety‐ and accessibility‐related barriers impairing participation was particularly pronounced among early‐career participants (see Table 4). Four of the nine respondents who disclosed safety concerns and five of the eight who reported accessibility concerns were ECPs.

**TABLE 4:** The perception of safety and accessibility barriers impacting participation at the ESRS congress.

	Safety concerns (*n* = 97)		Accessibility concerns (*n* = 79)	
	Yes (*n* = 9)	No (*n* = 88)	Yes (*n* = 8)	No (*n* = 71)
ECPs	4	52	5	39
Mid‐career	1	5	0	6
Senior career	0	7	0	6
Career stage not specified	4	24	3	20

*Note:* Safety concerns correspond with the decision to attend and accessibility concerns relate to difficulties attending.

Abbreviations: Career stage not specified, participants did not indicate the stage of their career; ECPs, student, early‐career clinician, early‐career postdoc; Mid‐career, mid‐career clinician, mid‐career postdoc; Senior, senior career clinician, senior career professional, retired.

#### 3.2.3 Supervisory and Institutional Support

Participants expressed a range of supervisory and institutional support, from strong encouragement and full funding to a complete lack of mentorship in sleep research/medicine. Positive examples included supervisors proofreading abstracts, logistical assistance and providing funding for conference registration fees, travel and accommodation. Conversely, some respondents reported not having ‘a mentor from the sleep field’ (R014) and that their supervisors were not ‘interested in sleep research, which resulted in a lack of funding and limited guidance’ (R072).

#### 3.2.4 Flexible and Hybrid Formats

Suggestions to broaden inclusivity and accessibility included virtual oral presentations, pre‐recorded talks, interactive ePosters with QR linked videos and virtual poster halls. One participant (R049) also advocated for switching to a virtual presentation in situations such as unplanned or sudden illness (‘Virtual participation for sickness instead of changing the presenting author’) or last‐minute constraints and to provide more control over presentation slot timing. Across subgroups, participants endorsed hybrid and alternative presentation formats to bolster inclusion, particularly for those with health, caring or financial constraints.

Quantitatively, the perceived benefit of language support and virtual participation options was particularly pronounced among ECPs (see Table 5). Nine respondents (9.2%) indicated that language support would aid participation, four of whom were ECPs and 56.6% of those who reported virtual participation as beneficial for attendance (*n* = 83) were ECPs.

**TABLE 5:** The perception of language support impacting participation at the ESRS Congress and the benefits of virtual participation.

	Language support aid participation (*n* = 97)			Benefits of virtual participation (*n* = 93)	
	Yes (*n* = 9)	Maybe (*n* = 21)	No (*n* = 67)	Yes (*n* = 83)	No (*n* = 10)
ECPs	4	16	36	47	6
Mid‐career	1	1	4	6	0
Senior career	0	1	6	6	1
Career stage not specified	4	3	21	24	3

*Note:* Language support is in relation to aiding participation or attendance at an ESRS congress and the benefits of virtual participation refer to whether virtual presentation modes would facilitate attendance for those who face in‐person constraints.

Abbreviations: Career stage not specified, participants did not indicate the stage of their career; ECPs, student, early‐career clinician, early‐career postdoc; Mid‐career, mid‐career clinician, mid‐career postdoc; Senior, senior career clinician, senior career professional, retired.

Respondents, particularly ECPs, consistently framed conference participation as central to career advancement, networking and equitable representation. Dedicated awards, recognition opportunities and early career‐specific events were suggested to enhance visibility and support progression in their fields (‘Offer more “Rising Stars” or similar awards to early‐career researchers in several domains’ [R019]). Beyond formal recognition, other benefits of conference participation included ‘network opportunities and new ideas’ (R002), exposure to ‘cutting edge science’ (R001) and visibility at early career sessions and award platforms. Nevertheless, structural barriers to equitable participation were also identified. Some reported perceptions of gatekeeping, whereby ‘big names and known names get the talks’ (R089) and that ‘membership advantages are not visible’ (R011).

Most respondents (*n* = 63; 69.2%), regardless of career stage, indicated that not attending the ESRS Congress would have a moderate to significant impact on their career progression (see Table 6). Specifically, 29 (31.9%), 11 (12.1%) and 23 (25.3%) participants rated non‐attendance as having a moderate, major and significant influence, respectively, with ECPs accounting for the largest proportion of these responses (62.1%, 63.6%, 47.8%, respectively). Ten respondents (11.0%) indicated non‐attendance as having no impact and 18 (19.8%) reported minor impact.

**TABLE 6:** Impact of non‐attendance at ESRS congress on career advancement.

	Level of impact on career advancement (*n* = 91)				
	None (*n* = 10)	Minor (*n* = 18)	Moderate (*n* = 29)	Major (*n* = 11)	Significant (*n* = 23)
ECPs	4	12	18	7	11
Mid‐career	0	1	1	1	2
Senior career	2	2	3	0	0
Career stage not specified	4	3	7	3	10

*Note:* The extent to which not attending an ESRS Congress would negatively impact participants' career progression stratified by career stage.

Abbreviations: Career stage not specified, participants did not indicate the stage of their career; ECPs, student, early‐career clinician, early‐career postdoc; Mid‐career, mid‐career clinician, mid‐career postdoc; Senior, senior career clinician, senior career professional, retired.

Further subgroup analyses to identify the specific barriers to conference attendance and their ranked importance among participants who had submitted an abstract that was not accepted and consequently did not attend the ESRS Congress (*n* = 8), stratified by career stage, are presented in Table 7. ECPs (*n* = 3) reported childcare, breastfeeding, logistical challenges, travel/accommodation costs, lack of travel funding or support and conference registration fees as highly important reasons for non‐attendance (impact score 5). One mid‐career respondent similarly highlighted travel/accommodation costs as most important. One senior‐career participant reported their abstract not being accepted as the paramount reason for non‐attendance, followed by conference registration fees (impact score 3) and visa‐related issues (impact score 2). Those whose career stage was unspecified (*n* = 3) noted travel/accommodation costs, lack of reduced fees, lack of travel funding or support, conference registration fees and personal reasons as highly important reasons for non‐attendance and their abstract being rejected as slightly important (impact score 2).

**TABLE 7:** Barriers to attending an ESRS Congress and their ratings of the importance of their impact for participants who submitted an abstract that was not accepted.

	ECPs (*n* = 3)	Mid‐career (*n* = 1)	Senior career (*n* = 1)	Career stage not specified (*n* = 3)
Childcare	5 (*n* = 1)			
Breastfeeding	5 (*n* = 1)			
Logistical challenges (e.g., travel restrictions, scheduling conflicts)	5 (*n* = 1)			
Travel/accommodation costs	5 (*n* = 1)	5 (*n* = 1)		5 (*n* = 1)
Lack of reduced fees				5 (*n* = 1)
Lack of travel funding or support	5 (*n* = 1)			5 (*n* = 1)
Conference registration fees	5 (*n* = 1)		3 (*n* = 1)	5 (*n* = 2)
Abstract not accepted			5 (*n* = 1)	2 (*n* = 1)
Visa‐related issue			2 (*n* = 1)	
Personal reasons				5 (*n* = 1)

*Note:* Impact scores range from 1 to 5, where 5 reflects the most important reason and 1 represents not at all important for attending the ESRS congress for participants who submitted an abstract that was rejected. ‘*n*’ reflects the number of participants within the specified career stage that indicated the given rating.

Abbreviations: Career stage not specified, participants did not indicate the stage of their career; ECPs, student, early‐career clinician, early‐career postdoc; Mid‐career, mid‐career clinician, mid‐career postdoc; Senior, senior career clinician, senior career professional, retired.

## 4 Discussion

This study highlights that barriers and facilitators surrounding in‐person Sleep Europe conference attendance vary considerably, with diverse experiences reported across different groups. The findings demonstrate that participation is shaped not solely by individual motivation, but by structural, financial and organisational conditions that influence who can attend and subsequently benefit from international scientific meetings. Organising the discussion according to the identified themes clarifies both the persistence of barriers and the scope of existing and potential society‐level responses.

### 4.1 Financial and Structural Barriers

Financial constraints emerged as the most frequently reported barrier, particularly among ECPs and researchers based in lower‐resource or economically unstable settings. Registration fees, travel costs, accommodation expenses and visa application charges collectively limited attendance, consistent with previous research (Velin et al. 2021; Raby and Madden 2021). These structural costs disproportionately affect students, trainees and researchers from LMICs (Bradley et al. 2020; Hutchins and Berkenstock 2025), reinforcing inequities in academic visibility and career progression (Humphries et al. 2021).

Visa‐related challenges were found to further exacerbate geographic disparities. Delays, refusals and high application costs resulted in non‐attendance despite accepted abstracts, echoing concerns raised in broader analyses of international academic mobility (Sarabipour et al. 2021; Doğan et al. 2023). The combination of quantitative patterns and qualitative accounts builds on insights from existing investigations (Mair et al. 2018), underscoring that conference participation is impacted by structural inequities across career stages, institutions and countries (Podreka et al. 2024).

Participants suggested that supportive financial strategies should be stratified by career stage and national context to bolster conference attendance. Microgrants for visa costs, local transport and differential registration structures were frequently highlighted as relevant avenues to achieve this. These suggested measures align with the ESRS's ongoing efforts to support participation through grants, fellowships and other initiatives, detailed in Table S1.

### 4.2 Accessibility, Disability and Safety Needs

Accessibility‐related barriers were prominent across qualitative accounts from all subgroups, whereby caregiving responsibilities, breastfeeding needs, disability accommodations and hearing accessibility influenced attendance decisions (McClurg 2024). Safety concerns related to the conference location were also reported, particularly among researchers travelling from abroad (Page and Mosen 2024).

Recommended practical measures included breastfeeding facilities, clearer caregiver support policies, live captioning and embedded accessibility planning within conference design. Despite ESRS Sleep Europe Congresses routinely providing breastfeeding rooms alongside prayer rooms, our findings suggest that these may not be clearly advertised to participants, which has implications for their decision to attend such events. While many societies, including the ESRS, offer measures such as childcare support during conferences, accessibility is most effective when treated as a structural component of conference planning rather than an optional add‐on (McClurg 2024). The ESRS has taken significant steps to improve equity, diversity and inclusion (EDI) at its events. One key initiative is the caregiver grant, originally launched as a childcare grant by the GEF and expanded in 2026 to be more inclusive of all caregiving responsibilities. This grant was positively received, demonstrating the impact of dedicated funding on improving accessibility and support measures for participants with caregiving responsibilities and expanded in 2026 to be more inclusive of all caregiving responsibilities.

In addition, the ESRS Equality Inclusion Diversity Policy and Code of Conduct for Events reflect another major step forward towards achieving a more inclusive, respectful and accessible conference environment. For Sleep Europe 2026, participants will be required to agree to follow the ESRS Code of Conduct for Events at the time of registration, highlighting the society's commitment to creating a safe and inclusive environment for all attendees. These measures complement broader efforts to reduce structural barriers and enhance participation across diverse subgroups.

Embedding accessibility, safety and inclusivity as explicit criteria in conference planning frameworks may reduce exclusion and improve equitable participation (Serrato Marks et al. 2021). Guidance on planning inclusive and accessible events emphasises that including accessibility from the earliest stages of event design allows for meaningful engagement for all attendees (Doran et al. 2024).

### 4.3 Supervisory and Institutional Support

Participants underscored the relevance of supervisory encouragement and institutional financial support for attendance, reinforcing existing evidence that organisational support plays a central role in conference participation (Wenger and Stauffacher 2025; Sancheznieto et al. 2024). Participants who received abstract feedback, proofreading support, logistical assistance and dedicated travel budgets directly from their institutions were substantially more likely to attend the ESRS Sleep Congress. The frequency of these enablers being reported was particularly pronounced among ECPs, suggesting that those early in their career rely heavily on supervisory and institutional support to facilitate participation in international sleep conferences.

Conversely, a lack of mentorship in the sleep field, absence of departmental travel funds and limited institutional advocacy were cited as direct reasons for non‐attendance (Weissgerber et al. 2020). This suggests that when institutional resources are limited, particularly in relation to financial assistance, initiatives adopted and promoted by scientific societies may enable ECPs to overcome such institutional barriers (Segarra et al. 2020).

### 4.4 Flexible and Hybrid Formats

The shift towards hybrid formats during the COVID19 pandemic demonstrated the feasibility of alternative participation models (Goodale et al. 2026). Moving academic conferences online has been shown to reduce economic, physical and legal barriers that have traditionally restricted participation (Levitis et al. 2021). Flexible and hybrid conference structures were identified as important facilitators for individuals facing visa‐, caregiving‐, financial‐ or health‐related barriers. In the sleep research community, initiatives such as eSleep, an online conference organised by the ESRS every second year, which is offered free of charge for its members, illustrate how fully virtual formats can broaden participation.

Although in‐person networking remains highly valued, hybrid participation may serve as a structural equaliser, particularly for researchers in lower‐resource settings or those unable to travel due to visa restrictions or caregiving responsibilities. Hybrid formats can increase access to scientific content, presentations and events (Guetter et al. 2022); yet they may also limit opportunities for building and strengthening professional networks (Estien et al. 2021). Therefore, sustained incorporation of flexible participation pathways may represent a key equity strategy rather than a temporary pandemic adaptation (Brohus et al. 2024).

### 4.5 Career Advancement, Inclusivity and Equity

Beyond immediate logistical barriers, participants emphasised the importance of conferences for visibility, networking and long‐term career progression, aligning with prior research findings (Spann et al. 2025). Structural exclusion from scientific meetings risks compounding disadvantage, particularly for ECPs and researchers in LMICs (Sidney 2019). We found that awareness and visibility of these opportunities were not universal, as ECPs reported that access to information about funding schemes and participation support was limited. Targeted outreach to ensure harder‐to‐reach populations (e.g., researchers in LMICs and those outside established academic networks) are aware of available events and opportunities may bolster equitable participation, ensuring that support mechanisms (e.g., travel awards, grants and mentoring programs) reach those who stand to benefit most.

Importantly, equity strategies must move beyond the availability of grants alone. Ongoing reviews of funding levels, targeted outreach to underrepresented regions and explicit inclusion policies are necessary to ensure that conference participation reflects the diversity of the global professional sleep community. For Sleep Europe 2026, the ESRS explicitly considered EDI criteria in the symposia selection, demonstrating a concrete approach to embedding inclusivity within the scientific programme. The efforts of the ESRS towards embedding EDI within the scientific programme may serve as a model for other societies to strengthen equitable participation, ensuring accessibility, safety and inclusivity are also core criteria in conference planning.

### 4.6 Strengths and Limitations

A key strength of this study is its mixed‐methods design, capturing both the prevalence and lived experience of those faced with conference attendance barriers. Importantly, this is the first study to specifically explore conference participation barriers among ECPs within the sleep field. Furthermore, the international recruitment strategy yielded a globally representative sample of sleep clinicians and researchers. Finally, the explicit focus on ECPs and detailed measurement of employment status, mobility, caregiving responsibilities and accessibility needs provides a nuanced picture of how structural conditions shape conference participation.

Several limitations should be acknowledged. The overall sample size, while adequate for mixed‐methods survey work, represents a fraction of the European sleep community and may limit the precision of geographic‐based subgroup estimates. Despite an effort to recruit through several European networks and societies, participation from some countries and regions remained low, which may underrepresent contexts where barriers are most acute. Furthermore, although 254 individuals initiated the survey, only 97 completed it. As participation was voluntary, the final sample may not be fully representative of all respondents and completion may have introduced selection bias (Shiyab et al. 2023). Additionally, a few participants reported a rejected abstract and non‐attendance, limiting the generalisability of findings from this group. Self‐selection bias may have also been present, whereby individuals with more recent or salient experiences of barriers might have been more inclined to respond than those without such experiences. Finally, the primary focus on the ESRS and closely related conferences may limit the relevance of our findings to other disciplines. Nevertheless, many identified barriers, such as costs, visas, accessibility and safety have been identified in global health (Velin et al. 2021), tourism and hospitality (Mair et al. 2018) and sport management (Yamashita and Oshimi 2023) conferences, among several others, demonstrating that these identified challenges may be shared and the corresponding solutions transferable across scientific disciplines.

### 4.7 General Recommendations for More Equitable and Inclusive Conferences

- *Promote Initiatives*: Increase visibility of grants, fellowships and hybrid participation options, particularly for ECPs.
- *Inclusive & Accessible Spaces*: Plan congress venues with hearing loops, quiet/sensory‐friendly spaces and adequate seating to reduce crowding.
- *Targeted Outreach*: Channel information to researchers from LMICs, underrepresented regions and ECPs with tailored communication and support.
- *Ongoing Evaluation*: Monitor uptake of equity measures and adjust strategies to maximise participation and inclusivity.

## 5 Conclusion

This study found financial, visa and institutional barriers to hinder ECPs' participation in international sleep conferences. Additional considerations such as accessibility requirements, language support and safety for minority groups intersect with structural barriers, influencing participation experiences. Organisations, including the ESRS, often provide travel grants, childcare support, hybrid participation options, breastfeeding rooms and prayer spaces to enhance attendance. Nevertheless, to further increase participation and meet the diverse needs of the professional sleep community, greater visibility, wider advertisement, flexible application timelines and increased grant availability are warranted. Given the diverse experience of participation barriers reported by distinct subgroups, including ECPs and those from LMICs, conference stakeholders should continue providing targeted support to promote equitable access and ensure such measures are visible. Ongoing evaluation and tailored strategies are key to allow for all participants to have meaningful opportunities for scientific exchange, networking and visibility.

## Author Contributions

**Monisha Ravikumar:** conceptualization, project administration, writing – original draft, writing – review and editing, data curation, methodology. **Michaela Pawley:** investigation, data curation, formal analysis, writing – original draft, writing – review and editing. **Anna M. Biller:** conceptualization, methodology, writing – review and editing. **Lieve van Egmond:** investigation, resources, writing – review and editing. **Laura Stankeviciute:** investigation, resources, writing – review and editing. **Matias Rusanen:** conceptualization, methodology, writing – review and editing. **Daniela Noain:** conceptualization, methodology, writing – review and editing. **Inês Dias:** methodology, writing – review and editing. **Iuliana Hartescu:** conceptualization, methodology, software, writing – review and editing. **Fran Pilkington‐Cheney:** methodology, writing – review and editing, conceptualization, resources. **Adriana Michalak:** visualization, project administration, supervision, writing – review and editing, conceptualization.

## Funding

The authors have nothing to report.

## Conflicts of Interest

The authors declare no conflicts of interest.

## Supporting information


**Table S1:** ESRS Provision Box: Grants, Fellowships and Initiatives to Promote Participation Across Themes (2012–2025).

## Data Availability

The data that support the findings of this study are available in the Loughborough University Research Data Repository at https://doi.org/10.17028/rd.lboro.31136236. Due to privacy and ethical restrictions, the dataset is under embargo and is not publicly accessible. Access to the data may be considered upon reasonable request and subject to approval by the relevant ethics and data governance procedures.
