# Supplementary material for: Participation in Sleep Europe Conferences: Insights From Early Career Professionals
Source: J Sleep Res. 2026 May 31;35(5):e70378. doi: 10.1111/jsr.70378 (PMC13619041; doi:10.1111/jsr.70378)
Supplement: Supplementary file 1 [file JSR-35-e70378-s001.docx]

**Supplementary Material**

**Table S1:** *ESRS Provision Box: Grants, Fellowships, and Initiatives to Promote Participation Across Themes (2012–2025)*

| **ESRS Initiative** | **Description** | **Year Established** | **Number Awarded per Year (Max)** | **Funding / Notes** | **Source** |
| --- | --- | --- | --- | --- | --- |
| **Financial Support** | | | | | |
| **Sleep Europe Sustainable Travel Grants** | Available for presenting authors | 2024 | 10 | €500 each | <https://esrs.eu/sleep-congress/> |
| **Childcare Grant** | Childcare grant, established for Sleep Europe 2022. Developed into the 2026 Care and Accessibility grant to account for other caring responsibilities and EDI issues | 2022 | 10 | €500 each | <https://esrs.eu/grants-awards/sleep-europe-childcare-support-grant/> |
| **Meetings & Courses Fellowship** | Support travel and accommodation for ESRS-endorsed events | 2017 | Up to 6 | €500 each | <https://esrs.eu/grants-awards/meetings-and-courses-fellowship/> |
| **Short-Term Research Fellowship** | Support cross-institutional collaboration and mobility | 2016 | 1–6+ | €2,800 each | <https://esrs.eu/news/2024-esrs-grants-fellowships-open/> |
| **Two-Week Training Grant** | Short-term research mobility funding for early-stage professionals | 2016 | 1 | €1,400 each | <https://esrs.eu/grants-awards/two-week-training-grant/> |
| **Sleep Europe Travel Grants (Abstract-linked)** | Reduced registration fees for ECPs; differential pricing for students/trainees | 2012+ | Variable | Variable | <https://esrs.eu/sleep-congress/abstracts/> |
| **Sleep Medicine Examination Grant** | Covers the ESRS Sleep Medicine Examination fee; includes one copy of the ESRS Sleep Medicine Textbook | 2022 | Up to 6 | € 250 each | <https://esrs.eu/grants-awards/sleep-medicine-examination-grant/> |
| **Paediatric Sleep School Grant** | Support attendance at the ESRS Paediatric Sleep School; includes accommodation and catering | 2024 | 10 | €500 each | <https://esrs.eu/paediatric-sleep-school/> |
| **Sleep Science School Grant** | Covers registration fee for the ESRS Sleep Science School, including meals and accommodation | 2021 | 10 | €500 each | <https://esrs.eu/sleep-science-school/> |
| **Accessibility & Inclusion Measures** | | | | | |
| **Hybrid Congress Format (Sleep Europe)** | Hybrid participation options allow both in-person and virtual attendance | 2020 (expanded during COVID) | Congress-wide | Enables remote participation for those facing financial, visa, caregiving, or mobility barriers | [esrs.eu](https://esrs.eu/esleep-europe/) |
| **eSleep Europe (Virtual Congress Platform)** | Fully virtual scientific programme with live and on-demand sessions | 2023, 2025 | Congress-wide | Provides flexible access to scientific content without travel | [esrs.eu](https://esrs.eu/esleep-europe/) |
| **On-Demand Session Recordings** | Recorded sessions available post-congress | Following each Sleep Europe congress | Congress-wide | Supports asynchronous participation across time zones | [esrs.eu](https://esrs.eu/esleep-europe/) |
| **Early Career Day** | Dedicated networking sessions for Early Career Professionals | 2012+ | Sleep Europe-based | Promotes inclusive engagement and professional integration | [esrs.eu](https://esrs.eu/) |
| **Accessibility Statements in Congress Materials** | Information outlining accessibility provisions | 2018+ | Congress-specific | May include venue accessibility info and inclusion policies | [esrs.eu](https://esrs.eu/) |
| **On-Site Childcare Support** | Physical facilities such as a breastfeeding room | Congress-specific | Congress-specific | Caregiving attendees | [esrs.eu](https://esrs.eu/) |
| **Hybrid & Flexible Participation** | | | | | |
| **Flexible Registration Categories** | Alternative registration models for hybrid/virtual participation | 2020+ | Congress-specific | Supports attendance for remote or constrained participants | [esrs.eu](https://esrs.eu/) |
| **Hybrid Congress Formats (general)** | Expanded hybrid sessions and online accessibility | 2020–2022 | Congress-wide | Facilitates remote participation and broader inclusion | [esrs.eu](https://esrs.eu/esleep-europe/) |
| **On-Demand Session Recordings** | Recorded sessions for asynchronous access | 2020+ | Congress-wide | Supports accessibility and flexibility | [esrs.eu](https://esrs.eu/esleep-europe/) |
| **Equity-Oriented Initiatives** | | | | | |
| **Differential ECP Registration Fees** | Reduced fees for students, trainees, and ECPs | 2012+ | Congress-specific | Encourages participation from early-career and underrepresented groups | [esrs.eu](https://esrs.eu/) |
| **Travel Grants Prioritising Presenting Authors** | Supports career visibility for accepted abstract presenters | 2012+ | Variable | Promotes equity in conference participation | [esrs.eu](https://esrs.eu/) |
| **Early Career Network Representation in ESRS Governance** | EC voices included in society’s decision-making | 2022+ | Ongoing | Promotes inclusive governance and representation | [esrs.eu](https://esrs.eu/) |
| ***The European Sleep Research Society has implemented a range of supportive initiatives, enhanced visibility, targeted stratification and periodic evaluation of these provisions may further strengthen equitable access*** | | | | | |

**Supplemental Information**

The questionnaire and recruitment strategies for the study are publicly available via the published workflow ([Ravikumar et al., 2026](https://doi.org/10.17028/rd.lboro.31353709)).

**Participation in the ESRS Conferences: Challenges for Early Career Professionals**

The archived dataset currently appears as:

Ravikumar, Monisha; Pawley, Michaela; Biller, Anna; van Egmond, Lieve; Stankeviciute, Laura; Rusanen, Matias; et al. (2026). Questionnaire for Participation in the ESRS Conferences: Insights from Early Career Professionals. Loughborough University. Workflow. <https://doi.org/10.17028/rd.lboro.31353709.v2>
